# Psychological testing in the profession of psychology: an Australian study

**DOI:** 10.1080/00049530.2024.2419682

**Published:** 2024-11-08

**Authors:** Peter Macqueen, Jo-Anne M. Abbott, Nigar G. Khawaja, Rebecca Mathews, Douglas Scott, Bruce D. Watt

**Affiliations:** a Compass Consulting, Australia; b Australian Psychological Society, Australia; c Queensland University of Technology, Melbourne, Australia; d Australian College of Applied Professions, Australia; e Monash University, Australia; f Bond University, Australia

**Keywords:** Area of practice endorsement, Australia, psychologists, testing attitudes, testing survey, training

## Abstract

**Objectives:**

In Australia, psychological measurement and testing is a core competency for all registered psychologists. This study aimed to provide a current perspective on the views of Australian psychologists in the use of psychological testing given the lack of recent Australian research.

**Method:**

Psychologists (*N* = 821) completed online a demographic form and the European Federation of Psychologists’ Associations (EFPA) questionnaire on Test Attitudes of Psychologists – Modified (EQTAP-M), refined for the Australian setting. Constructs addressed in the survey included test appreciation, training, technology-based testing, attitudes to test use, and self-rated competence. The sample mean age was 50 years, with 84% over 34 years and 20% being males.

**Results:**

Attitudinal differences, based on demographics and practice endorsement area, were examined. Partial support was obtained for the factorial model of previous EFPA studies. There was no difference in attitudes based upon the gender of the psychologists, but older psychologists reported less favourable attitudes and lower competence for psychological testing. Psychologists holding endorsements in organisational psychology, neuropsychology, and educational and developmental psychology indicated more positive testing attitudes and appreciation than other areas of practice. The application of technology in assessment was identified as an area that warrants further investigation and training.

**Conclusions:**

The outcome has theoretical and practical implications for professional bodies and educational institutions that develop policies and training programs relevant to psychological measurement and testing.

## Key Points

**What is already known about this topic:**

1. Psychological testing competence is a requirement for Australian psychologists.
2. A broad survey of Australian psychologists’ views on the use of psychological tests has not been undertaken for decades.
3. Published European studies on test use have revealed mixed results, and data are more than 10 years old.

**What this topic adds:**

1. This study shows that psychological testing is a core activity for at least a small proportion of Australian psychologists.
2. Based on the Australian sample, only partial support is provided for the factorial model revealed in the European studies.
3. Psychologists’ views on the value of testing, and the impact of technology, vary across certain demographics and psychologists’ area of practice.

The development and application of psychological tests has been considered a significant achievement of psychology in the 20^th^ century (Zimbardo, 2004). Many countries make extensive use of psychological and educational tests and Australia is no exception, with a rich history of psychological and educational test development and use (O’Gorman et al., 2022; Shum & Kinsella, 2012). The regulatory body for psychologists, the Psychology Board of Australia (PsyBA), has defined eight core competencies for general registration as a psychologist in Australia, with “Conducts psychological asssessments” featuring as competency number four in the revised professional competencies to take effect from December 2025 (Psychology Board of Australia, 2024). This competency includes 4.5 “Carries out, scores and interprets assessment measures.” and 4.6 “Formulates results of the assessment measures.”

Similarly, the Australian Psychology Accreditation Council (APAC), the Government appointed accrediting authority for the training of psychologists in Australia, has developed accreditation standards for psychology programs requiring the development of psychological testing competencies, including standards for graduates of psychology programs in general, and for the nine Areas of Practice Endorsement (AoPE). An Area of Practice Endorsement recognises a psychologist as having advanced training in an area of psychological practice approved in the National Law (PsyBA, 2019). Furthermore, the report on the International Project on Core Competences in Professional Psychology (International Project on Competence in Psychology, 2016) identifies psychological testing and evaluation as a core competence for psychologists and has been adopted internationally, including by the Australian Psychological Society (Australian Psychological Society, 2018).

Psychological testing is an essential aspect of counselling and clinical care (Afolabi, 2015). Valid tests are used in a standardised manner to gather pertinent information to conceptualise and diagnose the client and to plan and execute the intervention. The American Psychological Association (American Psychological Association, APA Task Force on Psychological Assessment and Evaluation Guidelines, 2020) Guidelines for Assessment and Evaluation under the heading “Avoidance of harm”, emphasise that “Psychological testing, assessment, and evaluation is a core component of psychological practice, treatment planning, and subsequent decisions regarding those served” (p. 5). Test data is used to make critical decisions not only in clinical and counselling settings, but also in educational, organisational, neuropsychological, developmental, forensic, sport, medical and legal settings (Frenkel & Butkowsky, 2020; Krutko et al., 2018). Considering the vital role of psychological testing, psychologists are guided by professional standards of practice to take utmost care when engaging in psychological testing, assessment, and evaluation. The APA 2020 Guidelines urge psychologists to be compliant with competency expectations and to avoid harm to clients.

The profession of psychology in Australia has undergone significant transformation over the past two decades. With changes in the Australian government health system, psychologists are preferring to enter private practice. The number of members of the APS reporting their primary employment setting as independent practice having almost doubled in the decade following the introduction of Medicare in 2006 (Mathews, 2018).

It is important to note that most government funding for psychological services is directed to psychologists in private practice for intervention services. The psychologists can claim their clients’ treatment-related fees through the Australian Medicare system. However, the Australian Medicare system does not reimburse psychologists for formal psychometric assessment. Subsequently, there is a trend to engage in the treatment of clients (which is reimbursed) and to refrain from assessment and testing (which is not reimbursed).

These changes raise questions about the participation of psychologists in assessment services, particularly when formal psychometric testing, traditionally, has formed an important part of diagnoses and evaluations. This shift might also raise questions about service integrity given that testing is seen to be essential to therapeutic treatment services both in the planning and decision-making and also in the ongoing management of clients through the use of evidence-based outcome measurement (American Psychological Association, APA Task Force on Psychological Assessment and Evaluation Guidelines, 2020; Faija et al., 2022; Kantrowitz et al., 2018). This system driven development has also resulted in a change to the training programs for psychologists to accommodate the increasing shift towards treatment services and away from psychometric testing. This move away from training in psychometric assessment has been documented both within (Gonsalvez et al., 2021) and outside of Australia (Curry & Hanson, 2010; Merenda, 2007) and it has been argued in other jurisdictions that similar reasons underpin this shift (see Eisman et al., 2000).

The practice of psychological testing has also had a major transformation. Contemporary practice is more mindful of equity in psychological testing, with consideration for example of bias, cultural appropriateness, accommodations, and notions of validity beyond a straightforward metric (Rust et al., 2021; Shum et al., 2017). Camara and Merenda (2000) highlight the importance of considering a range of technical and professional issues, including job analysis, discrimination guidelines and test choice, in an employment setting. Further, technology is having an impact on the development and use of psychological and educational tests (Nye, 2022). Landers and Behrend (2023) provide insight into the impact of predictive models developed by artificial intelligence and the range of perspectives that can be taken by different professionals engaged in modern assessment. Clearly, stakeholders, such as educational institutions, government service providers, professional psychological societies, and professional regulators need to be engaged actively within the psychology-testing-technology domains, including addressing emerging professional and ethical issues. The rapid increase in technological developments is likely to challenge the traditional role of psychologists (Iliescu & Greiff, 2019) and these developments are only part of the changing relationships with stakeholders in the testing ecosystem (Iliescu & Ispas, 2018).

These contemporary developments and changes to testing practices highlight the need to better understand the use of psychological tests as well as the focus of training in psychological test use for psychologists in Australia. No recent information about psychological test use in Australia is available. Sharpley and Pain (1988) published an account of general test usage which was restricted to tests drawn from the 1983/84 catalogue of the Australian Council of Educational Research (ACER), with respondents providing ratings against test use frequency, value in practice, and recommendations for inclusion in training courses. Consistent with subsequent European studies, the Wechsler scales were ranked very highly on each of these criteria. A more recent survey focussed specifically on tests used by psychologists for court purposes finding neuropsychological tests being the most used instruments (Martin et al., 2007).

International surveys have been conducted (e.g., Evers et al., 2017; Wright et al., 2017), several of which included analysis of test attitudes of psychologists using a tool developed by the European Federation of Psychologists’ Associations (EFPA) Standing Committee on Tests and Testing and the International Test Commission (ITC), the EFPA Questionnaire on Test Attitudes of Psychologists (EQTAP).

Studies about these surveys reported on data collected in 2012 or prior. Evers et al. (2017) focussed primarily on European respondents but did include data from New Zealand as one of the 29 participant groups in the 2012 collection, but data from Australia were not collected. Although the profession of psychology shares common principles globally, there is variation in the training pathways and the regulation through policies and procedures. Thus, there appears to be a clear gap in understanding of contemporary psychological testing practices, attitudes and the training of psychologists in test use in Australia.

Given the significance of testing to psychology practice, the changes to psychological testing highlighted here and the potential gap in current knowledge of test use amongst the psychology profession, the Australian Psychological Society (APS) Tests and Testing Expert Group (TTEG) initiated a survey of Australian psychologists with a view to addressing a range of issues associated with the use of psychological tests by psychologists in Australia. While the initial aim in developing the survey was to address local issues, the inclusion of the EQTAP was seen as an opportunity to incorporate an instrument with solid psychometric properties and with published studies based on a large sample of overseas psychologists, producing findings of interest against which the Australian study could be compared. For example, Evers et al. (2017) noted significant differences between clinical, educational, and organisationally oriented psychologists on some of the five scales formulated on the basis of an Exploratory Factor Analysis. Compared to clinical psychologists, psychologists providing organisational services had a less stringent view on the regulation of tests and testing, and a more positive view towards internet testing and its value. Nevertheless, across 29 countries there was a “widespread lack of enthusiasm” (p. 183) for internet testing. The authors suggested that these findings, based on 2012 data, may have reflected the overall age of the respondents (mean = 41.4 years), and that internet testing was much more prevalent in organisational settings than clinical and educational settings. Male psychologists were more inclined to hold a favourable view towards internet testing and less stringent views regarding regulation. This pattern, to some extent, was replicated across age groups and the three areas of practice.

The current study adopted an expanded version of the EQTAP survey by gathering more specific information on the psychologist test user and the context of their psychology services. Thus, this comprehensive 2021–22 survey of Australian psychologists aimed to identify the attitudes, opinions, training, and challenges associated with the current use of psychological tests, and to determine if such views converged or differed across psychologists working in different settings and across AoPE. Utilising the EQTAP provided the opportunity to compare contemporary Australian data with previous findings.

## Method

### Participants

There were 821 participants in the study with 92% (*n* = 759) being members of the Australian Psychological Society (APS) and 8% (*n* = 62) psychologists who were not APS members. The majority were female (78.5%), while a small number (1.5%) identified as other. Most (72%) of the participants were aged between 35 and 64 years, while 14% were aged between 25 and 34 years, 15% were over 64 years and only 0.2% were under 25 years. Over half (52.3%) of the participants had a Master’s degree, while others had completed a PhD (20.9%), DPsych (14%), Honours (6.5%), Bachelors (2.2%) and other degrees (4.1%). Duration of working as a psychologist ranged from not having worked professionally yet (i.e., currently studying) to 58 years (Mean = 17.3 years). Over half (57%) of the participants identified themselves as endorsed in clinical psychology, while other endorsements held^1^ were in clinical neuropsychology (17.1%), educational and developmental psychology (15.6%), organisational psychology (9%), counselling psychology (7%), health psychology (6.1%), forensic psychology (4.3%), community psychology (1.6%) and sport and exercise psychology (0.5%). Participants were primarily practising in Victoria (33.3%), New South Wales (27.4%) and Queensland (22.5%), with smaller numbers from Western Australia (8.4%), South Australia (5.2%), Australian Capital Territory (4.4%), Tasmania (4%) and Northern Territory (1.5%). The majority (70.4%) of participants worked in major cities, while others worked in regional (23%), rural (5%), and remote (1.6%) areas. They identified themselves as mostly working in the private (65.3%), or public (28.1%) sectors or for other non-government/not for profit agencies (6.6%). Approximately one-third (35%) of participants reported that most of their paid work time involved psychological testing.

### Measures

#### Demographic questionnaire

The demographic questionnaire captured the participants’ age, gender, highest qualification, duration of work in psychology, location, setting, sector of main work role, and any areas of practice endorsement. Background and contextual information captured about psychological testing included the percentage of work time spent in psychological testing, populations tested, types of tests administered, test accommodations, platforms used to conduct assessments, digital testing skills, training and professional development needs, and the impact of COVID-19 on testing.

#### EFPA Questionnaire on Test Attitudes of Psychologists (EQTAP; Evers et al., 2017)

The EQTAP consists of 25 items. The response option for most items uses a 5-point Likert Scale (1= totally disagree to 5 = totally agree), with one open-ended item eliciting the name of the three tests most frequently used. Questionnaire items were found to comprise a five-factor solution. The factors were i. *Concerns over incorrect test use* (8 items), ii. *Regulations on tests and testing* (8 items), iii. *Internet testing* (6 items), iv. *Appreciation of tests* (4 items) and v. *Knowledge and training relating to test use* (3 items). In the European study the Cronbach’s alphas for internal consistency for factors i, ii, iii, iv, and v were .88, .66, .68, .70 and .56, respectively.

The present study adapted the EQTAP to suit the purpose of the research and to ensure relevance to the Australian context (see Table 1 for items). A new item about the APS, the professional body for psychologists in Australia was added to the *Regulation on test and testing scale* along with one additional item on internet testing. Two items were added to the *Test appreciation* factor. Two of the original items were retained for the *Knowledge and Training* factor and five new items were added to this cluster. A new theme was developed for this study which was identified as *Competence: self-rating*, with 5 items. The final version (EQTAP-M) resulted in 34 Likert scale items, 14 of which were modified from the original EQTAP.

**Table 1.** Confirmatory factor analysis loadings and Cronbach’s alpha for revised EQTAP-M.

	Initial λ	Final λ
Test Appreciation α = .77		
(1) I use tests regularly as part of my psychological services.	.572	.570
(2) Tests constitute an excellent source of information if they are combined and complemented with other psychological data.	.886	.888
(3) Used correctly, tests are of great help to the psychologist.	.922	.927
(4) In the last decade tests and testing practices have improved in Australia.	.340	.335
(5) Psychological tests rarely add information beyond a well-conducted interview (R).	.467	.445
(6) The time and expense required for administration, scoring and interpreting outweighs any real benefits (R).	.549	.531
Training Attitude α = .78		
(1) My training has been sufficient for the correct use of most tests.	.754	.749
**(2) My current knowledge about tests is what I learned as a part of my tertiary education.**	**−.016**	**NA**
(3) I have adequate resources to extend my knowledge or use of tests.	.626	.619
(4) I have attended professional development related to testing and assessment in the past 2 years.	.537	.550
(5) My training has not adequately prepared me for test administration, test interpretation and report writing (R).	.624	.620
**(6) I require professional development events to extend my testing skills.**	**−.327**	**NA**
(7) I avoid psychological testing because I question my knowledge and skills to complete competent assessments (R).	.758	.757
Technology α = .80		
(1) Test administration over the Internet has many advantages compared with paper-and-pencil administration.	.750	.735
(2) Computer-generated interpretive reports do not have any validity (R).	.441	.408
(3) Test administration over the Internet sets some test takers at a disadvantage (R).	.406	.394
(4) If properly managed, the Internet can greatly improve the quality of test administration.	.744	.822
(5) The privacy of the test taker is not protected when testing by Internet (R).	.614	.556
(6) nTesting over the Internet opens the way to fraud (R).	.562	.557
(7) The nature of Internet-based testing limits the value of online assessments (R).	.724	.767
Attitudes to Test Standards α = .77		
(1) The International Test Commission or another international organisation should establish a global system to accredit the certification of test users.	.393	.446
(2) The use of psychological tests should be restricted to qualified psychologists.	.563	.516
(3) National guidelines [e.g., APS Ethical guidelines for psychological assessment and the use of tests, Practice guide for the use of psychological tests and instruments] should be promoted strongly.	.596	.607
(4) Standards [e.g., those of the European Federation of Psychologists’ Associations (EFPA), or of the American Psychological Association (APA)] defining the minimum technical qualities of a test should be enforceable	.596	.655
(5) Legislation is needed to control serious abuses of testing.	.571	.608
(6) Anyone who can demonstrate their competence as a test user (whether a psychologist or not) should be allowed to use tests (R).	.451	.344
(7) Control on tests and testing should be minimal, as control discourages the development of new ideas and new procedures (R).	.587	.499
(8) Test publishers should be allowed to sell whatever tests they think fit (R).	.473	.392
(9) The Psychology Board of Australia should take a more active role in the regulation and improvement of test use.	.567	.593
Competence α = .84		
(1) I use a range of psychological tests competently (recognised psychological tests rather than checklists).	.833	.834
(2) I have sound skills to interpret formal tests.	.927	.927
(3) I have satisfactory skills to interpret a range of psychological tests.	.859	.859
(4) I can prepare high-quality reports based on tests.	.860	.860
(5) I have mastered tests delivered in digital format.	.403	.404

**Bold** = not included in final scale analyses. (R) = (Reversed).

### Procedure

Ethics approval was obtained from the Griffith University Human Research Ethics Committee. Six psychologists with extensive knowledge about assessment were invited to review the survey measures which contributed to the refinement of the questions. The study was disseminated to members of the APS through the organisation’s official email newsletter and social media platforms. A weblink took participants to the information sheet, consent form and questionnaires. They were informed about confidentiality and their right to withdraw from the research at any point. Further communication about the study was published via LinkedIn (both individual and groups) and a snowballing technique was used with the research team emailing the weblink to their professional networks. This led to a small increase in the sample size, including some non-APS members. The demographic profile of participants compared reasonably well with demographic data available from the Psychology Board of Australia about all registered psychologists in Australia. The survey took approximately 15–20 min to complete with the data collected between November 2021 and June 2022.

### Data analysis and design

This paper is one of a series of potential papers reporting on the analysis of a broad range of data collected using this comprehensive survey instrument. This paper specifically reports on the responses to the Likert-scaled attitudinal items. Results from other sections of the survey, such as perceived training needs and identification of the most used tests, are presented in Mathews and Khawaja (2024).

A Confirmatory Factor Analysis (CFA) was used to examine the factor structure of EQTAP-M. The CFA was used to examine the structure of the data considering the earlier European results, and the dimensions or factors included as combined scales were considered more reliable than scales considered separately. Bivariate correlations and MANOVAs were used to examine whether there were differences on the subscales of EQTAP-M for gender, age, years of experience, and educational qualification. Focussing upon the work of the psychologists, MANOVAs were conducted to test for differences on the EQTAP-M subscales by psychologist qualifications and AoPE. Statistically significant MANOVAs were followed up with univariate ANOVAs for each of the five EQTAP-M subscales.

## Results

CFA was completed for the survey items using SPSS version 26.0 and maximum likelihood analysis. Model cut-offs for acceptable solutions were specified as < .08 for root mean square error of approximation (RMSEA), >.90 for Tucker-Lewis index (TLI) and > .90 for comparative fit index (CFI) (Finch & French, 2019). The initial CFA was run with all items and the five factors as specified by Evers et al. (2017). Covariance between all factors demonstrated acceptable fit, with RMSEA = .065, but below acceptable for TLI = .82 and CFI = .84. Examination of the item standardised regression weights identified two items with loadings < .30, which were removed from further analyses (see Table 1). As identified in the modification indices, there was significant covariance between the error components for reverse scored items.

CFA was repeated with two poorly performing items removed. The two items removed were both clustered under the dimension “Training”. Error covariances were added for the reverse scored items. The adjustments improved the model fit identifying an acceptable index for RMSEA = .060, though outside the .90 cut-off for TLI = .866, CFI = .879. Standardised item regression weights presented in Table 1 show loadings above .40 for all items except four items which met a lower threshold of .30: Appreciation 4, Technology 3, Standards 6, Standards 8. Cronbach’s alpha indicated acceptable internal consistency for research purposes for the subscales and high internal consistency for the Competence subscale. Descriptive analyses identified substantial negative skew for the subscales Appreciation, Training, Standards, and Competence. Analyses were repeated using reverse scoring with Log (10) transformation to reduce potential errors. The findings were mostly the same, with any exceptions outlined below.

### The effects of demographics on the attitudes toward psychological testing

Initial analyses evaluated the effects of demographics and work contexts in predicting percent of time spent performing psychological testing and psychologist’s test attitudes. No gender differences were found for percent of time spent testing, *t* (714), *p* = .09, or the five psychological test attitudes scales, *F* (5, 710) = 0.57, *p* = .72, *η*^2^ = .004. Psychologist age was significantly correlated with Appreciation *r* (713) = −.11, *p* < .001, Technology *r* (713) = −.22, *p* < .001, Training Attitude *r* (713) = .10, *p* =.01, and Competence *r* (713) = −.14, *p* < .001. This means that older psychologists reported less favourable attitudes towards psychological testing and perceived themselves as less competent compared to younger psychologists. A similar finding was obtained for the correlation between years of practice and test attitudes, statistically significant for Technology *r* (713) = −.20, *p* < .001, Appreciation *r* (713) = −.10, *p* =.006, and Competency *r* (713) = .09, *p* =.02. Percent of time spent undertaking psychological testing was inversely related to age, *r* (711) = −.12, *p* = .001, but unrelated to years of practice, *r* (713), *p* =.11.

The MANOVA indicated Test attitudes did not differ according to educational qualifications *F* (10, 1420) = 1.58, *p* = .108, *η*^2^ = .011 but, univariate between-subjects effects reflected statistically significant differences for Technology *F* (2, 713) = 4.30, *η*^*2*^ = .012. Psychologists with master’s level qualifications reported more favourable attitudes towards technology use compared to doctoral level qualifications. A significant finding was obtained for the Log (10) transformed data for Competence, *F* (2, 713) = 3.36, *p* = .035, *η2* = .009 with doctoral qualified psychologists reporting higher perceived competence compared to bachelor and honours qualified psychologists. Psychologists’ educational qualification was unrelated to the percentage of time spent testing, *F* (2, 713) = 0.71, *p* = .50

Participant responses for work context formed 10 categories including community and family (*n* = 25), disability/rehabilitation (*n* = 22), education (*n* = 101), health (*n* = 80), mental health (*n* = 29), organisational (*n* = 51), university and research (*n* = 43), private practice solo (*n* = 172), private practice team (*n* = 155), and other (*n* = 38). A MANOVA conducted on test attitudes reflected a statistically significant difference across work contexts *F* (45, 3530) = 2.51, *p* < .001, η^*2*^ = .031. Test of between-subject effects was statistically significant for the Technology scale *F* (9, 706) = 7.01, *p* < .001, η^*2*^ = .082, with participants working in an organisational context reporting more favourable attitudes compared to all other participants, and team private (health) practitioners providing higher ratings compared to psychologists working in public health. A significant between-subjects effect was obtained for Competence *F* (9, 706) = 2.93, *p* =.002, η^*2*^ = .036, with psychologists in organisational contexts providing higher scores compared to private practice solo and the other category. This finding should be considered with caution, as the result was not statistically significant with the Log (10) transformation analysis. The between-subjects effect was significant for Appreciation, *F* (9, 706) = 3.05, *p* = .001, η^*2*^ = .037, though none of the post hoc analyses were statistically significant. An absence of significance was found for attitudes towards Training, *F* (9, 706) = 0.95, *p* = .094, η^2^ = .021, and Standards, *F* (9, 706) = 0.25, *p* = .606, η^2^ = .010.

Differences across work contexts were found for percent of time involved in psychological testing by work context, *F* (9, 706) = 3.86, *p* < .001, η^2^ = .047. Post hoc analyses indicated that psychologists working in university and research described lower proportion of time involved in testing compared to those working in community/family and health settings. Psychologists working in sole private practice reported less time spent testing compared to practitioners in health. No differences were found between the remaining categories.

In evaluating differences across the factors for AoPE, and using dummy variable coding for AoPE, initial analysis compared participants with an AoPE to respondents without an AoPE. Next, the analyses were completed for each AoPE, so that participants who reported a particular AoPE (e.g., Clinical Psychology) were compared to all other respondents (e.g., all participants without a Clinical Psychology AoPE including those with another AoPE and those without any AoPE). MANOVAs are reported with the five test survey attitude scores as the dependent variables, and where these are statistically significant, univariate test of between subject effects (ANOVAs) are reported for the significant differences between the groups. T-tests were conducted to examine differences in percent of time spent in psychological testing for each AoPE. Due to the small but statistically significant effect for age, post hoc analyses were completed, testing for the interactions for each AoPE x age for each factor. None of the interactions were statistically significant, thereby the results are not included in this paper. Data for those endorsed in Community and Sports and Exercise psychology was removed from the analyses due to the small sample size (*n* = 9 and 3, respectively).

Comparing participants with any AoPE to respondents without an AoPE, a statistically significant MANOVA was found for Test Attitudes *F* (5, 710) = 2.40, *p* =.036, η^2^ = .017. The univariate test of between subject effects indicated a statistically significant difference for Appreciation *F* (1, 308) = 5.45, *p* = .02, η^*2*^ = .008. Psychologists reporting any AoPE reported lower appreciation for testing compared to non-AoPE (see Table 2). There was no difference for the remaining four test survey scales.

**Table 2.** Means and standard deviations for scores on the five factors by area of practice endorsement (AoPE).

AoPE		Appreciation		Training		Technology		Standards		Competence	
	*N*	*M*	(*SD*)	*M*	(*SD*)	*M*	(*SD*)	*M*	(*SD*)	*M*	(*SD*)
Any	492	4.19	0.61	4.00	0.77	3.25	0.61	3.90	0.55	4.12	0.73
No	224	4.31	0.51	4.02	0.73	3.29	0.62	3.82	0.55	4.10	0.62
Neuro	88	4.46↑	0.66	4.51↑	0.44	2.84↓	0.49	4.06↑	0.55	4.55↑	0.40
Clinical	274	4.11↓	0.65	3.82↓	0.78	3.25	0.59	3.92	0.51	3.99↓	0.73
Counselling	32	4.03	0.65	3.82	0.89	3.30	0.42	3.75	0.47	3.90	0.95
Ed/Dev	81	4.36↑	0.45	4.29↑	0.59	3.19	0.67	3.94	0.51	4.27↑	0.58
Forensic	23	4.25	0.70	4.20	0.63	3.09	0.65	3.83	0.58	4.30	0.55
Health	28	4.06	0.46	3.60↓	0.86	3.32	0.57	3.60↓	0.56	3.74↓	0.87
Org	45	4.26	0.42	4.09	0.68	3.60↑	0.60	3.89	0.68	4.36↑	0.56

Neuro = Clinical Neuropsychology. Ed/Dev = Educational Developmental. Org = Organisational. ↑ = p < .05 with higher scores. ↓= *p* < .05 with lower scores.

A statistically significant MANOVA was obtained for clinical neuropsychology, *F* (5, 710) = 23.59, *p* < .001, η^*2*^ = .142. The univariate test of between subject effects was statistically significant for all subscales, Appreciation *F* (1, 714) = 15.03, *p* < .001, η^2^ = .021, Training *F* (1, 714) = 47.58, *p* < .001, η^2^ = .062, Technology *F* (1, 714) = 50.42, *p* < .001, η^2^ = .066, Standards *F* (1, 714) = 11.32, *p* < .001, η^2^ = .016, and Competence *F* (1, 714) = 42.18, *p* < .001, η^2^ = .056. Psychologists with clinical neuropsychology endorsement reported greater test appreciation, more favourable attitudes towards training and test standards, and higher self-perceived competence compared to psychologists without clinical neuropsychology endorsement. In contrast, lower ratings for technology were provided by endorsed clinical neuropsychologists compared to other psychologists.

The MANOVA was statistically significant for AoPE clinical psychology, *F* (5, 710) = 7.72, *p* < .001, η^2^ = .052. Statistically significant univariate between subject effects were obtained for Appreciation, *F* (1, 714) = 19.93, *p* < .001, η^2^ = .027, Training, *F* (1, 714) = 25.79, *p* < .001, η^2^ = .035, and Competence, *F* (1, 714) = 14.33, *p* < .001, η*2* = .020. Endorsed clinical psychologists reported lower appreciation of psychological testing, less favourable view of training, and lower self-reported competence compared to other respondents. A similar pattern of MANOVA results was obtained for the AoPE of health psychology, *F* (5, 710) = 3.08, *p* =.009, η^2^ = .021, with univariate significant effects for Training, *F* (1, 714) = 8.31, *p* =.004, η^2^ = .012, Standards, *F* (1, 714) = 7.59, *p* =.006, η^2^ = .011, and Competence, *F* (1, 714) = 8.64, *p* = .003, η^2^ = .012. Endorsed health psychologists reported less favourable attitudes towards training, standards, and competence compared to other psychologists.

Significant MANOVA results for endorsed educational and developmental psychologists, *F* (5, 710) = 2.98, *p* =.011, η^2^ = .021, reflected univariate statistically significant between subject effects for Appreciation, *F* (1, 714) = 4.33, *p* < .038, η^2^ = .006, Training, *F* (1, 714) = 13.19, *p* < .001, η^2^ = .018, and Competence, *F* (1, 714) = 4.85, *p* =.028, η^2^ = .007. Of note, Appreciation was not statistically significant using the Log (10) transformed data. Educational and developmental psychologists reported greater appreciation for psychological testing, more favourable attitudes towards training, and greater self-perceived competence in comparison to other psychologists.

The statistically significant MANOVA results for endorsed organisational psychologists, *F* (5, 710) = 4.33, *p* < .001, η^*2*^ = .03, reflect a similar pattern of results as for respondents who reported working in an organisational context. Statistically significant univariate between-subject effects were obtained for Technology, *F* (1, 714) = 5.40, *p* < .001, η^*2*^ = .02 and for Competence, *F* (1, 714) = 2.85, *p* = .015, η^2^ = .008. These findings indicate greater appreciation for online assessment and technology along with greater self-reported competence for psychological testing compared to other respondents. The MANOVAs were not statistically significant for AoPEs in counselling *F* (5, 710) = 1.20, *p* =.30, η2 = .008, or forensic psychology, *F* (5, 710) = .1.00, *p* =.42, η^2^ = .007. The analyses comprised small numbers for each AoPE thereby limiting the power for the analyses.

In evaluating proportion of work allocated to psychological testing, neuropsychologists and educational/developmental psychologists reported greater time spent conducting assessments, while clinical, counselling, and health psychologists reported less time spent testing.

## Discussion

Psychological testing is seen as a cornerstone of psychological practice, and the application of the principles of psychometrics has long been considered essential in contributing to the evidence by which a practitioner will formulate an evaluation (Vitoratou & Pickles, 2017; Zimbardo, 2004), therefore representing a critical component of practice in psychology. This Australian study investigated the current use of psychological tests, psychology training in test use, and the attitudes of psychologists to testing practices. In addition, the study explored differences in test use that may exist across practice settings and AoPE.

In undertaking the study, we adopted a measure supported in international research (EQTAP) and tailored this to the Australian context (EQTAP-M). The modified scale represented five dimensions. The findings, to some degree, replicate findings of recent international studies using this measure that noted some resistance to online testing as well as the influence of workplace setting on psychometric test use (e.g., Evers et al., 2017). In the current study attitudes towards testing, engagement in test use, and perceptions of competence varied across several demographic variables, although there were no significant differences found in the perceptions of competence when considering the gender of psychologists. The results highlighted differences in attitudes associated with age, training, and the setting in which psychological testing was undertaken, as summarised in the following three sections.

## Age

The findings demonstrate less favourable attitudes towards psychological testing generally, and in particular regarding internet and technology-based testing, as the age of the psychologist increases. This may be associated with several factors including discomfort with a changed test environment or testing platform, changes in the mix of professional activities undertaken, or reduced time in undertaking testing during a period of partial employment. Differential attitudes based on age (or years of experience) could be explored in a separate manuscript by drawing on the qualitative data from this survey.

## Qualifications and training

While psychologists with postgraduate training reported more favourable attitudes towards testing when compared to those psychologists without postgraduate training, those who held a master’s degree demonstrated the most positive attitudes to psychological testing, even higher than those with a more advanced doctoral-level training. It may be that this is a result of the more practically focussed training that occurs in a professional master’s program as opposed to more research-based doctoral degrees. It seems that for some psychologists with postgraduate qualifications, psychological testing appears to be more dominant in their training and their practice. Nevertheless, those with doctoral degrees reported more perceived competence. This apparent discrepancy between perceived competence and attitudes to psychological testing would require investigation beyond the realms of this study.

When inspecting the different endorsed areas of practice, which can only be achieved through professional postgraduate training (most commonly a master’s but may also be a professional doctorate), psychologists endorsed in neuropsychology, educational/developmental, and organisational psychology reported more favourable attitudes towards psychological testing than those with other AoPEs. This is not unexpected as neuropsychologists in Australia have a strong focus on clinical assessment and similarly organisational psychology very much involves testing, albeit focused more on performance and recruitment. Educational and developmental psychologists have an emphasis on appraising factors impinging upon learning in school and tertiary contexts. Psychologists with clinical, counselling, and health AoPEs reported less involvement in psychological testing, while clinical and health reported less favourable attitudes towards the use of tests, training and competence in psychological testing, perhaps reflecting greater emphasis on providing therapeutic services than evaluation. Nevertheless, Mathews and Khawaja (2024) provide survey respondent data on perceived training needs in psychological testing, with 65% of respondents checking “New tests”, followed by “Test interpretation” (54%), “Technology enhanced testing” (44%), Test adaptation (40%), Report Writing (38%), and “Psychometrics” (32%).

## Work setting

While participants reported working in various settings including in education, health centres and universities, by far the most represented workplace setting was independent/private practice. A surprising result therefore was that 35% of participants spent most of their work time undertaking psychological testing. Indeed, over a third reported that psychological testing was a central aspect of their daily work, perhaps an unanticipated finding given the shift of psychologists in private practice services following Government funding for treatment services under Medicare (Mathews, 2018). It may be that in the context of such treatment services, psychological testing continues to remain a core aspect of psychology work. An alternative and more plausible explanation may be that the participant sample in the current study, which represents only a small number of registered psychologists in Australia (2.4% of registered psychologists at the time of data collection), may have selected to complete the survey because they were involved actively in psychological testing as part of their work.

Nevertheless, in the current sample some differences in test use and attitudes across work settings was found, as was the case with Evers et al. (2017), and the unpublished follow-up by Jimenez (2023). Psychologists working in an organisational setting as an employee held more favourable attitudes to psychological testing than those working in other settings, but this trend was not replicated with those holding an AoPE in organisational psychology in comparison to other AoPEs. However, the analyses for work settings and for AoPE involve overlapping groups. For example, 49% of organisational AoPE reported organisational as their work setting category, and the corresponding 51% for the same AoPE reported alternative work locations (such as university and research, or private practice (solo or team)). Conversely, of those total respondents who reported working in the organisational setting, 55.6% were organisational AoPE and 44.4% were not organisational AoPE. Consequently, the findings for work settings and for AoPE require separate interpretation.

However, these two overlapping groups (organisational AoPE and those respondents working in organisational settings) were the most appreciative of technology-based testing. Again, this is not unexpected given the many psychological tests used in an organisational setting, and certainly more than any other setting, are administered using an online platform (Australian Psychological Society, 2018). Echoing this technology uptake by organisations, Kantrowitz et al. (2018) found that of Australian and New Zealand firms using testing for recruitment, over 90% were conducted online, and 22% used mobile-based assessment. In addition, 79% of firms reported using testing for assessing senior managers and executives. Although likely a biased sample, given this was a vendor-based survey, all three percentages were the highest globally.

## Theoretical and practical implications

The results add to the literature by highlighting the attitudes towards psychological testing in the current professional and service context. It is interesting to note that many psychologists continue to use psychological tests in their daily work; however, some psychologists, primarily those with an AoPE in organisational psychology or clinical neuropsychology, hold more positive views on psychology test use and are more likely to use tests in the workplace. In addition, organisational psychologists have more positive attitudes and report higher levels of confidence in online and technology-based testing when compared to psychologists working in other contexts. In contrast, older psychologists reported lower perceptions of competence in such testing and were less likely to use these platforms or technologies in their work. The apparent differential perspectives on testing, particularly with respect to the use of technology, could be explored in combination with the qualitative data from this survey and via further targeted research.

The findings are important for professional bodies and educational institutions. Considering the vital role of test use in psychological practice, updating and revitalising the skills of psychologists in testing and assessment is important to ensure evidence-based practice and optimal outcomes for all stakeholders. Zickar et al. (2017) highlight the need for test users to consider not just test reliability and validity but also the availability and use of suitable test norms. This message aligns with the expression of interest of survey respondents in training in test adaptation, and the release of a comprehensive publication on test adaptation across linguistic and cultural situations (Iliescu, 2017). The International Test Commission (ITC), perceiving a need for further training for test users, recently introduced the ITC Learning Centre https://learning.intestcom.org/.

Professional development strategies appear to be warranted for older and more experienced psychologists as they reported lower perceived assessment competencies compared to their younger counterparts. Further investigation would need to be undertaken to establish the basis of this finding, but one possible explanation is a perception by older psychologists of skill decay (see also Arthur & Day, 2018; Arthur Jr et al., 1998); or skill gaps, particularly considering the rapid impact of new technology on the testing industry and testing practices.

More training is required to educate psychologists about the use, benefits, and issues associated with technologically assisted testing (Iliescu & Greiff, 2019). At the time of this survey development (2021) and data collection (2021–2022), the potential impact of artificial intelligence (AI) on psychological testing was not investigated explicitly, but this is likely to emerge as an important issue very soon. Generative AI is increasingly providing challenges and opportunities for psychologists in the provision of cost-effective, fair, valid and reliable resources to address the psychological issues confronted by clients across diverse domains. These recent developments are likely to have impacts also at the intervention level, with reports of ChatGTP-4 passing the Turing test (Jones & Bergen, 2024), and AI models possibly surpassing “humans” in the evaluation of constructs such as the perceived demonstration of empathy (Lee et al., 2024).

## Limitations and future direction

The present findings, despite being a national online survey, are limited and may not generalise to all registered psychologists in Australia. The sample is a small proportion of the psychologists working in Australia and future studies should focus on large samples that strategically cover all areas of psychologists in rural and urban settings across all states and territories. Though use of th*e EQTAP-M* allows future comparison of this data with psychology practice in other countries, future studies can use a more comprehensive battery to capture various aspects of the testing related attitudes. Although quantitative analysis is a good first step, follow-up qualitative data would assist in unpacking the attitudes of Australian psychologists to psychological testing and assessment. The ongoing advances and commercial investment in machine learning and large language models, and their subsequent impact on mainstream psychological testing practices, will need to be monitored.

## Data Availability

The data are not publicly available due to their containing information that could compromise the privacy of research participants.

## Note

1. Some participants held more than one area of practice endorsement.
